# Hormonal regulation of colour change in eyes of a cryptic fish

**DOI:** 10.1242/bio.20149993

**Published:** 2015-01-16

**Authors:** Helen Nilsson Sköld, Daniel Yngsell, Muhmd Mubashishir, Margareta Wallin

**Affiliations:** 1 Sven Loven Centre for Marine Sciences, Kristineberg, University of Gothenburg, SE-450 34 Fiskebäckskil, Sweden; 2 Department of Biomedicine, Kristianstad University, SE-291 88 Kristianstad, Sweden; 3 Department of Biological and Environmental Sciences, University of Gothenburg, SE-40530 Gothenburg, Sweden

**Keywords:** Erythrophores, Iris, Physiological colour change, Pigment, Melanophores, Camouflage

## Abstract

Colour change of the skin in lower vertebrates such as fish has been a subject of great scientific and public interest. However, colour change also takes place in eyes of fish and while an increasing amount of data indicates its importance in behaviour, very little is known about its regulation. Here, we report that both eye and skin coloration change in response to white to black background adaptation in live sand goby *Pomatoschistus minutes*, a bentic marine fish. Through *in vitro* experiments, we show that noradrenaline and melanocyte concentrating hormone (MCH) treatments cause aggregation of pigment organelles in the eye chromatophores. Daylight had no aggregating effect. Combining forskolin to elevate intracellular cyclic adenosine monophosphate (cAMP) with MCH resulted in complete pigment dispersal and darkening of the eyes, whereas combining prolactin, adrenocorticotrophic hormone (ACTH) or melanocyte stimulating hormone (α-MSH) with MCH resulted in more yellow and red eyes. ACTH and MSH also induced dispersal in the melanophores, resulting in overall darker eyes.

By comparing analysis of eyes, skin and peritoneum, we conclude that the regulation pattern is similar between these different tissues in this species which is relevant for the cryptic life strategy of this species. With the exception of ACTH which resulted in most prominent melanophore pigment dispersal in the eyes, all other treatments provided similar results between tissue types. To our knowledge, this is the first study that has directly analysed hormonal regulation of physiological colour change in eyes of fish.

## INTRODUCTION

An increasing amount of studies report that the eyes of animals are important not only for vision, but also that the eye colour is involved in signalling and communication. In humans, the power of flirting is undisputable and eye colour may be a trait partly under sexual selection (Frost, 2006; Sturm and Larsson, 2009), and eyes are also major visual targets for humans (Levy et al., 2013). Eyes of birds, frogs and fish are often spectacularly coloured, either similar to the colours and patterns of the body, but sometimes also in contrasting colours. A study on American kestrel birds showed that the colour of iris varied significantly within and among the individuals (Bortolotti et al., 2003). The authors found that the eye colour changed from brown to red-brown with age and that younger males had more red eyes than females. They also reported that birds exposed to polycarbonated bromide showed less colourful eyes and reduced reproductive success.

In fish, several species are able change colour of the eyes and certain eye colour patterns correlate with aggressive behaviors, stress and social status (Vera Cruz and Brown, 2007; Nilsson Sköld et al., 2013; Freitas et al., 2014). In the presence of a predator, guppies for example show blackening of eyes (Magurran and Seghers, 1991), and the flexible blue eye colour in stickleback males is an ornament that attracts females (McLennan and McPhail, 1989; Flamarique et al., 2013). The significance of the eye appearance among fish is further supported by the presence of caudal eyespots that mimics the head and masks the tail which reduced tail fin predation (Winemiller, 1990). Whether eye coloration changes during background adaptation as is known for the skin, is however still uninvestigated. Moreover, little is known about the regulation of eye colour change. In a recent study on *Tripterygion delaisi*, which has bright red-fluorescent eyes, KCl-treatment revealed that changes in eye coloration of this fish are achieved through pigment translocations within iris melanophores (Wucherer and Michiels, 2014). Except for this study, which is indicative of neuronal regulation of fish eye colour change, nothing is to our knowledge known regarding what hormones, transmitter substances and second messengers that can mediate physiological colour change in eyes. No study has to our knowledge investigated direct *in vitro* effects of hormones on eyes either. This is rather remarkably given the overwhelming amount of research on coloration and colour change in skin, as pointed out in a recent review (Nilsson Sköld et al., 2013).

In order to shed light on the regulation of colour change in eyes, we have here investigated this matter in sand gobies, *Pomatoschistus minutes*, collected from the wild. This species is a small and highly transparent fish that lives on sandy bottoms in shallow waters. Eyes of this fish are normally cryptic in coloration like rest of the body and *in vitro* experiments have shown that this fish can change colour both in the skin and on the inside (Nilsson Sköld et al., 2010). Kvarnemo et al. (Kvarnemo et al., 1995) describe a darkening of the eyes during mating behaviors of this fish, indicating an ability to change the eye colour, and our hypothesis is that the eyes may change colour also during background adaptation. Here, we have using sand goby investigated (1); effects of background adaptation on eye coloration, (2); hormonal regulation of physiological colour change in isolated eyes, focusing on melanophore pigment aggregation and dispersion, and compared the effects between eyes, skin and peritoneum.

## RESULTS

### Description of the sand goby eye

**Fig. 1.**
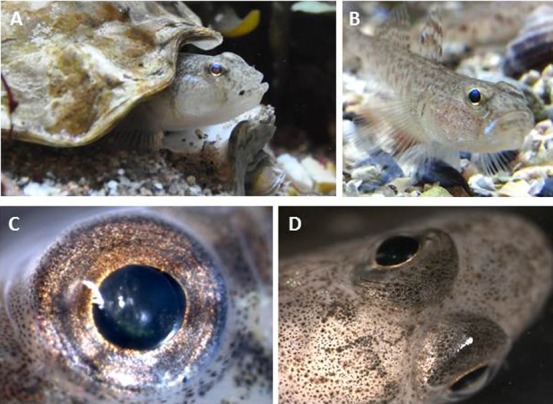
Eye coloration in living sand goby, *Pomatoschistus minutus*. (A) Photograph of a male sand goby guarding his nest. (B) Photograph of a sand goby on gravel. (C) Close-up micrograph of an eye *in situ* shows that the dorsal area is densely pigmented while the ventral side contains more reflective pigment. (D) Photograph taken from above the fish shows that the eye has a flat iris whereas the rest of the eye is spherical in shape and pigmented at the dorsal part. All photographs reveal cryptic eyes, similar in coloration to the rest of the head.

The sand goby eye has a flat and coloured iris that contains melanophores, iridophores and erythrophores with red/orange pigment. Iridophores are common, particularly at the ventral side of the eye, giving reflectance of the eyes (Fig. 1). Often three spots of large erythrophores and melanophores were observed within this commonly more pale ventral area (Fig. 1C). The dorsal area of the iris is often more pigmented. The rest of the eye is spherical in shape and although the iridophores dominate at the back of the eye, chromatophores of different pigment types are also frequent, especially at the dorsal side which is visible *in situ* to the observer (Fig. 1D).

### Background adaptation

**Fig. 2.**
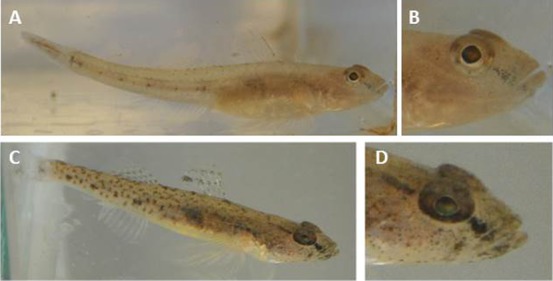
Background adaptation in living sand goby, *Pomatoschistus minutus*. Fish kept on a white background became considerable pale throughout the body and in the eyes (A,B), whereas the same fish showed darkening of the skin and eyes after subsequent transfer to dark background (C,D). B and D are magnifications of head area in A and C, respectively.

From visual observations of live fish and photographs, fish kept on a white background became considerable pale throughout the body and in the eyes (Fig. 2A,B). Pigment concentration into the ventral sparse chromatophores of the iris was apparent. After transfer to dark background, the same individuals showed darkening of the skin and eyes (Fig. 2C,D). The results were similar for all five replicates.

### Factors causing paling of eyes, peritoneum and skin

**Fig. 3.**
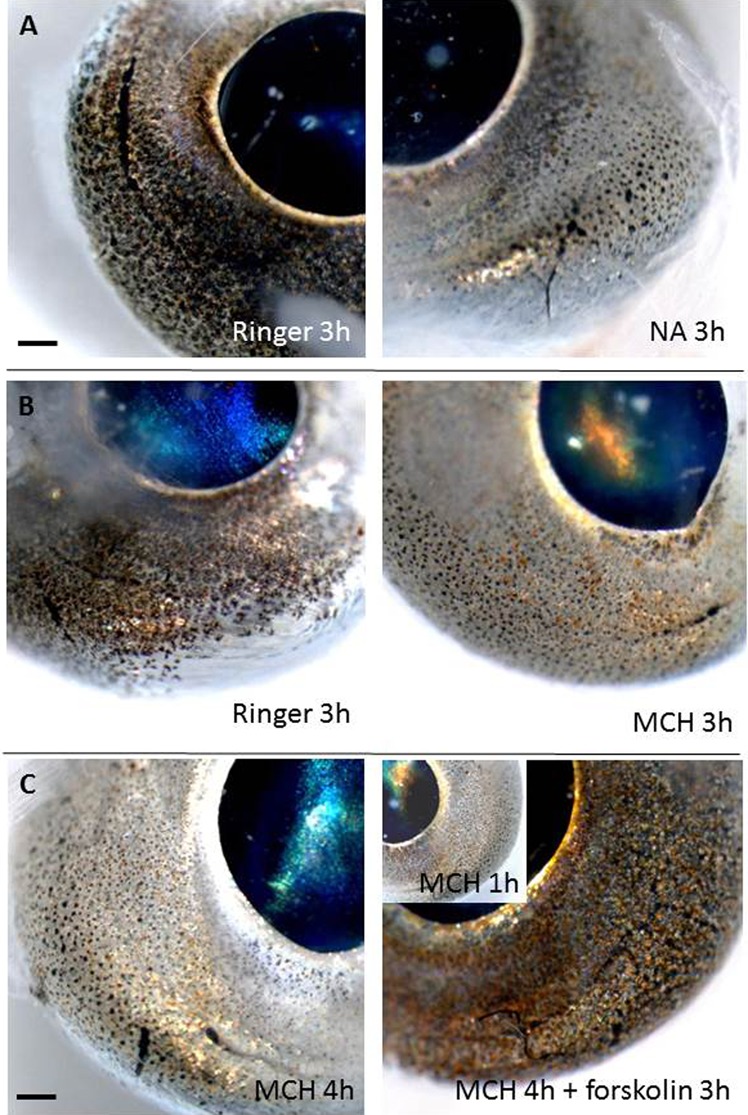
Physiological colour change *in vitro* in eyes of sand goby, *Pomatoschistus minutus*. Left panel are controls incubated in Ringers solution, whereas right panel is the hormone treated of the same eye pair with the results of pigment aggregation and paling of iris by MCH (A) and NA (B) after 3 h treatment. (C) The eyes were pre-incubated with MCH for 1 h to induce chromatophore pigment aggregation and then forskolin was added to one eye in the pair (right) with the results of pigment dispersal and darkening of the eye. Insert in C shows the MCH mediated paling of the eye before forskolin was added. Scale bars: 0.2 mm.

Both melanocyte concentrating hormone (MCH) and noradrenaline (NA) caused a rapid and persistent paling effect caused by pigment aggregation in melanophores and erythrophores of eyes, peritoneum and skin (Table 1; Fig. 3A,B). For MCH, the effect was significant already after one hour (eyes: P = 0.005, peritoneum: P = 0.025, skin: P = 0.003). The response to NA was similarly significant after one hour in eyes (P = 0.001) and peritoneum (P = 0.0001), but slower in skin melanophores and a significant effect was seen only after two hours (P = 0.028). Neither eyes, skin or peritoneum melanophores responded to light and the eyes remained dark (Table 1). It shall be noted that the skin melanophore pigment aggregated to some extent in the Ringer's solution during the treatment (Table 1).

**Table 1.**
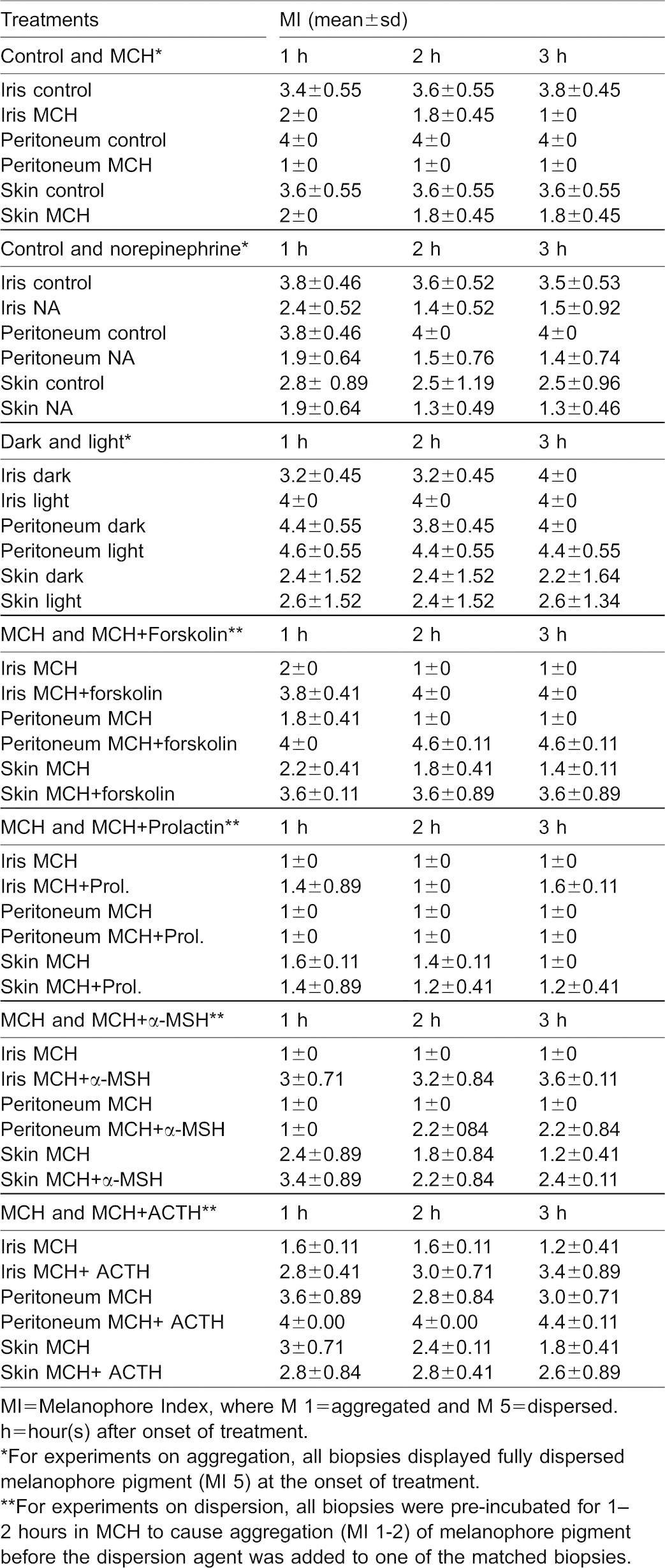
Physiological colour change responses in melanophores of eye, peritoneum and skin of sand goby

### Factors causing darkening or enhanced coloration of eyes, peritoneum and skin

**Fig. 4.**
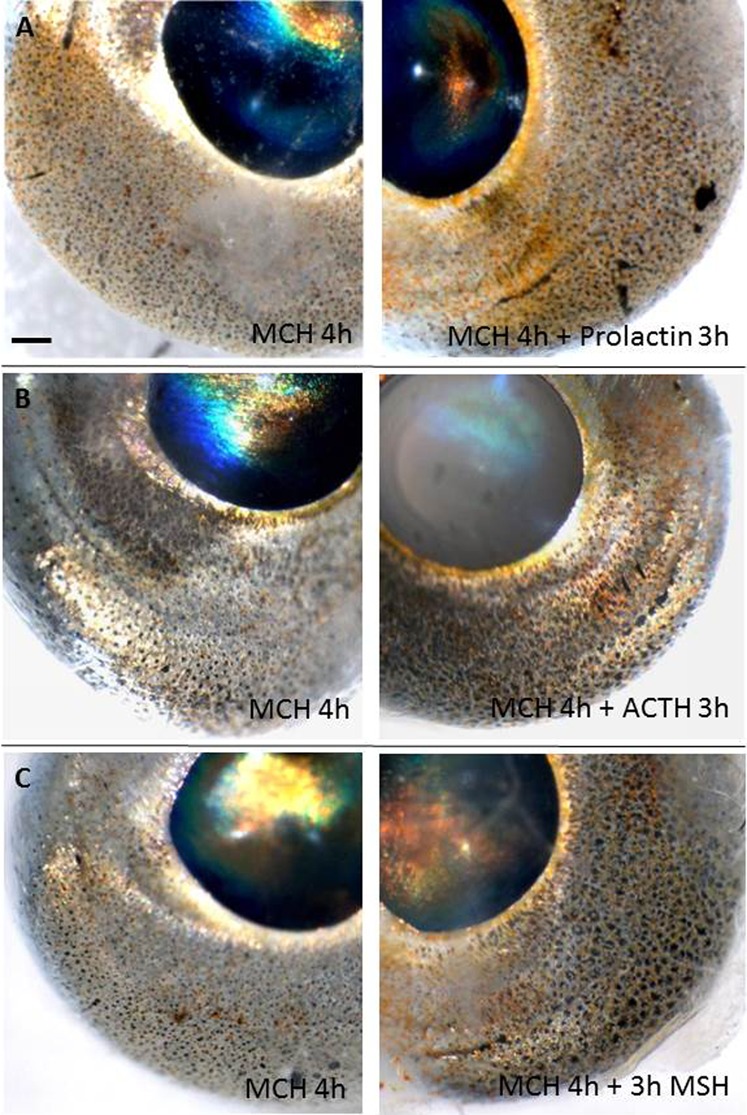
Physiological colour change *in vitro* in eyes of sand goby, *Pomatoschistus minutus*. Pigment dispersal effects of prolactin (A), ACTH (B) and α-MSH (C) after 3 h treatment. Left panel are controls incubated in MCH in Ringers solution, whereas right panel is incubated in MCH+the putative dispersal hormone for the same eye pair. Prolactin resulted in more orange coloration only compared to MCH only, whereas ACTH and α-MSH also caused melanophore pigment dispersal resulting in darker eyes. All eyes were pre-treated in MCH for at least one hour to induce pigment aggregation before addition of the dispersal hormone. Scale bar: 0.2 mm.

When forskolin was added to tissues pretreated with MCH, the eyes became considerable darker and melanophore pigment dispersed rapidly in eyes, peritoneum and skin (Table 1; Fig. 3C), indicating a role of cyclic adenosine monophosphate (cAMP) in eye and peritoneum pigment dispersal. The response was significant after one hour forskolin treatment (eye: P = 0.001, peritoneum: P = 0.0001, skin: P = 0.005). Prolactin had no effect on melanophores from either tissue, but gave a more yellow and red appearance of the eyes (Table 1; Fig. 4A) by dispersal of pigments in erythrophores/xanthophores. Both α-melanocute stimulating hormone (α-MSH) and adrenocorticitropic hormone (ACTH) caused pigment dispersal although less pronounced in the skin and peritoneum than in the eyes (Table 1; Fig. 4C,D). For α-MSH, the effect was significant in eyes (P = 0.003) and skin (P = 0.034) after one hour, but first after two hours in peritoneum (P = 0.033). ACTH dispersed melanophore pigment in the eyes after one hour (P = 0.005) and after two hours in peritoneum (P = 0.013), but had no dispersal effect on the skin melanophores. Even after three hours, there was no significant difference between MCH alone and MCH+ACTH in the skin (P = 0.11). It shall be noted, that MCH alone had a limited pigment aggregation effect of skin and peritoneum melanophores compared to the eyes in the ACTH experiment.

## DISCUSSION

Eyes of lower vertebrates are often spectacularly coloured and an increasing amount of data, indicate the importance of eye colour in communication and health. But while some fish species have the ability to change eye colour, remarkably little is known about its regulation. Using sand goby, we here show that both skin and eyes can adjust through paling or darkening responses for background adaptation. From *in vitro* experiments, we further show that the eye chromatophores are directly responsive to a range of hormones and factors previously known to mediate both physiological (pigment translocations) and more long-term colour changes in dermal chromatophores of many fish species (Sugimoto, 2002; Nilsson Sköld et al., 2013).

Our results show that elevating cAMP (by forskolin treatment) mediates melanophore pigment dispersal and darkening of the eyes, indicating that cAMP is likely to be a second messenger involved in eye chromatophore regulation, as it is for skin or scale melanophores and erythrophores (Thaler and Haimo, 1992). The hormones NA and MCH caused melanophore pigment aggregation and paling of the eyes while MSH and ACTH resulted in pigment dispersal and darkening of the eyes. Prolactin in combination with MCH had no effect on melanophores, but gave a more orange appearance of the eyes, presumably by dispersal of pigments in erythrophores/xanthophores as shown for dermal chromatophores in other fish species (Oshima et al., 1996; Nilsson Sköld et al., 2008). Light has previously been described to mediate pigment aggregation in cultured fish melanophores (Wakamatsu et al., 1980), but had no effect on any tissue melanophores of sand goby and the eyes stayed dark. However, incubation in dark may also result in melanophore pigment aggregation (Iga and Takabatake, 1983), but this was not seen in sand goby. Instead, control eyes or peritoneum incubated in Ringers only (and in dark) all displayed totally dispersed melanophores. Interestingly, iridophores present in the cornea of sand goby have earlier been described to change reflectance in response to light and dark (Lythgoe and Shand, 1989).

In a recent study of *Tripterygion delaisi*, a cryptic blenny which displays red fluorescent eyes, KCl-treatment caused eye colour change (Wucherer and Michiels, 2014). In the same study, histology of the fish iris revealed two layers of melanin containing cells, with the outer ones located among iridophores being responsive to KCl with pigment translocations. In the sand goby, not only melanophores and iridophores are present in the eyes. Responsive chromatophores containing red or yellow pigments are also clearly evident among the responsive melanophores.

By comparing colour change effects on eyes, skin and peritoneum of the same fish, our results indicate that the regulatory pattern is similar between the different tissues, which is relevant for the cryptic life strategy of this fish. In presence of predators, sand gobies burrow or stay in aggregates on the sand (Magnhagen and Forsgren, 1991). For camouflage and background adaptation in such situations, it makes sense to have a similar regulation of skin and eye coloration in order to obtain matching coloration. Also when the fish moves to different substrates or guard eggs in mussel shells, it is important that the eyes display an adjustable cryptic pattern and coloration that matches the background and the rest of the fish. Both NA and MCH resulted in melanophore pigment aggregation and paling of both eyes and skin. This result is consistent with other *in vitro* studies using skin or scales but also for analysis *in vivo*. Research on background adaptation in flounders for example, show that skin darkening correlates with higher plasma levels of α-MSH, whereas skin paling correlate with MCH and NA together with a reduction of pro-opiomelanocortin (Mizusawa et al., 2013) which is the prehormone to α-MSH and ACTH (Hadley, 1992).

Besides, sand goby females turn on a dark coloration around their eye openings and dark facial markings when they are ‘ready to mate’ (Kvarnemo et al., 1995; Forsgren, 1999; Pedroso et al., 2013). Because sand goby females are batch spawners, they are only interested in finding a male to spawn with when they have a mature clutch of eggs that is ready to be spawned. Hence, a female in this condition may together with a characteristic swimming behavior signals her intention to the males with the black markings around her eyes. With except for ACTH, all hormones and factors here tested *in vitro* resulted in similar response of the eyes and the skin. ACTH in the combination with MCH resulted in melanophore pigment dispersal in the eyes but not in the skin, why we anticipate that ACTH may be the mediator of dark eyes. Given the eye darkening effect of also α-MSH, this peptide shall however not be ruled out either.

In summary, this is to our knowledge the only study that investigated hormonal regulation of physiological colour change in eyes of fish. Given the importance of eye coloration in many different behaviors across species and that eye colour change may be a relatively common phenomenon among fish, we anticipate that our findings will stimulate further and more functional research on the largely neglected population of chromatophores that are located in the eyes. While our results on sand goby provide new and important clues on candidate hormones that could mediate the colour change in fish eyes, this can be followed up by *in vivo* studies for different species. By injecting fish with receptor blockers, it might be possible to reveal what factors, hormones and transmitters that mediate a colour change response in a specific behavioral situation. In species where the genome is sequenced, analysis of receptor transcripts levels are also possible (Mizusawa et al., 2013). In skin, long term (weeks) background adaptation or administration of hormones that stimulate physiological colour change also mediate morphological changes over time such as changes in chromatophore density and cell shape that aid the colour change (Sugimoto, 2002). Although it is possible that a constant long term physiological stimuli or cue will result in morphological colour change in eyes as well, this needs to be verified. The gap in knowledge also includes receptors and agonist specificities, deeper understanding of the intracellular pathways and mechanisms involved. Left to reveal is also how common colour change in eyes is among species, when it occurs and its overall role(s) for different species.

## MATERIALS AND METHODS

### Study object

Sand goby (*P. minutus*) at the size range of 4–5 cm used for the *in vitro* experiments were caught in May 2012, while fish used to assess effects of ACTH were caught in October 2013. Background adaptation experiments were performed in April 2014 with fish at the size range of 4.5–7 cm. All fish were caught using a ringnot in a shallow sandy bay at the mouth of the Gullmar fjord, Sweden (N 58°15′, E 11°27′). Following capture, the fish were transferred to and kept in glass aquaria with sand on the bottom and supplied with flow-through seawater under a natural night and daylight regime. Fish not sacrificed during the experiments were released back to the sea. Experiments conform to relevant regulatory standards concerning animal ethics. Ethical permission for the experiments was obtained from the Swedish animal welfare agency (245–2010, 205–2013).

### Background adaptation

Sand gobies (n = 5) kept on sandy bottoms were divided into two white buckets with aerated saltwater for two days for white adaptation, and then each fish was size measured to allow identification of each fish and photographed through a small glass aquarium under standardized procedures using a Nikon D90 camera (Nikon Nordic AB, Solna, Sweden). The fish were thereafter transferred to two black buckets for 3 days of dark adaptation under the same conditions as during white adaptation, then again size measured and photographed as earlier.

### Hormones and chemicals

Stock solutions of 1 mM NA (Sigma Aldrich, St. Louis, MO, USA) were stored at −20°C and diluted to the experimental concentration of 10 µM in Atlantic cod Ringer's solution at pH 7.5 (150 mM NaCl, 5.2 mM KCl, 1.8 mM MgSO_4_, 7.0 mM NaHCO, 1.9 mM NaH_2_PO_4_ and 1 mg/ml glucose), just before use. Sheep prolactin (Sigma Aldrich), were stored at −20°C as 150 IU/ml stock solution, and used at 0.150 IU/ml (corresponds to 5 µg/ml). α-MSH from ICN Pharmaceuticals, Inc. (Costa Mesa, CA, USA), was stored as 1 mM stock solutions at −20°C and used at 5 µM. MCH (Sigma Aldrich) was stored at −20°C as 10 µM stock solution and used at 100 nM. ACTH (Sigma Aldrich) was stored as 1 mM stock solution at −20°C and used at 1 µM. Forskolin (Sigma Aldrich) was stored in −20°C as 5 mM stock solutions made up in dimethyl sulphoxide (DMSO) and used at 5 µM.

The selected hormones were used because of their known effects on skin chromatophores in a range of fish species (Fujii, 2000; Nilsson Sköld et al., 2013) and the doses used are in line with previous work on physiological colour change in chromatophores using fish skin or scales (Sage and Bern, 1972; Ide, 1973; Logan et al., 2006; Nilsson Sköld et al., 2008).

### Colour change assay

Eyes and abdominal biopsies containing epidermal and peritoneal chromatophores were excised in filtered saltwater to give a pair of the different biopsies from each fish, and then placed in Atlantic cod Ringer's solution. The excised eyes displayed maximal dispersed chromatophore pigments in saltwater and in Atlantic cod Ringer's solution. The individual biopsies were then transferred into Nunc plastic wells (VWR International AB Stockholm) containing either 1 ml of Atlantic cod Ringer's solution (Nilsson Sköld et al., 2010), or NA or MCH freshly diluted into Atlantic cod Ringer's solution in order to induce pigment aggregation. The potential aggregating effect of light was investigated by keeping control biopsies in dark and their matched samples in daylight. In order to reveal regulation of dispersion, biopsies were pre-incubated in MCH for one to two hours before forskolin, prolactin, ACTH or α-MSH was added to one of the matched biopsies in the presence of MCH. The corresponding controls for the putative dispersing hormones are here the MCH incubated biopsies. DMSO (0.1%) was also included in the controls for the forskolin experiments.

The biopsies were incubated in dark and monitored paired wise after 1 h, 2 h and 3 h, post treatment. The grade of melanophore pigment aggregation or dispersal was scored manually under light microscopy by the use of the Melanophore Index, MI (Hogben and Slome, 1931) where fully aggregated pigment give MI = 1 and fully dispersed pigment give MI = 5. The chromatophores close to the lens of the eyes responded slower than the other chromatophores. For consistency, the dorsal area of the flat iris was used for scoring. Images were taken at the end of each experiment using a camera attached to a light microscope, both from Leica (Leica Microsystems AB, Kista, Sweden).

### Statistics

The *in vitro* experiment was designed for a paired two-tailed statistical test so that each fish had its own matched control eye and skin biopsy for each tested agent. A two tailed paired t-test or the non-parametric Wilcoxons signed rank test were used to statistically test the significance of a treatment in relation to its paired control at the same time point. The Kolmogorov-Smirnov-test was used for testing for distribution normality of the data. Statistical significance was set to P≤0.05 and all tests were run using the SPSS software.

### List of abbreviations

ACTH, adrenocorticotropic hormone; α-MSH, α-melanocyte stimulating hormone; cAMP, cyclic adenosine monophosphate; DMSO, dimethyl sulfoxide; MCH, melanocyte concentrating hormone; MI, melanophore index; NA, noradrenaline or norepinephrine.
